# Surface Properties and Photocatalytic Activity of KTaO_3_, CdS, MoS_2_ Semiconductors and Their Binary and Ternary Semiconductor Composites

**DOI:** 10.3390/molecules190915339

**Published:** 2014-09-24

**Authors:** Beata Bajorowicz, Anna Cybula, Michał J. Winiarski, Tomasz Klimczuk, Adriana Zaleska

**Affiliations:** 1 Department of Chemical Technology, Faculty of Chemistry, Gdansk University of Technology, ul. G. Narutowicza 11/12, Gdansk 80-233, Poland; E-Mails: beatabajorowicz@gmail.com (B.B.); anna-cybula@wp.pl (A.C.); 2 Department of Solid State Physics, Faculty of Applied Physics and Mathematics, Gdansk University of Technology, ul. G. Narutowicza 11/12, Gdansk 80-233, Poland; E-Mails: mwiniarski@mif.pg.gda.pl (M.J.W.); tomasz.klimczuk@pg.gda.pl (T.K.)

**Keywords:** composites, photocatalysis, semiconductor, tantalate, sulfide, pollutant degradation

## Abstract

Single semiconductors such as KTaO_3_, CdS MoS_2_ or their precursor solutions were combined to form novel binary and ternary semiconductor nanocomposites by the calcination or by the hydro/solvothermal mixed solutions methods, respectively. The aim of this work was to study the influence of preparation method as well as type and amount of the composite components on the surface properties and photocatalytic activity of the new semiconducting photoactive materials. We presented different binary and ternary combinations of the above semiconductors for phenol and toluene photocatalytic degradation and characterized by X-ray powder diffraction (XRD), UV-Vis diffuse reflectance spectroscopy (DRS), scanning electron microscopy (SEM), Brunauer–Emmett–Teller (BET) specific surface area and porosity. The results showed that loading MoS_2_ onto CdS as well as loading CdS onto KTaO_3_ significantly enhanced absorption properties as compared with single semiconductors. The highest photocatalytic activity in phenol degradation reaction under both UV-Vis and visible light irradiation and very good stability in toluene removal was observed for ternary hybrid obtained by calcination of KTaO_3_, CdS, MoS_2_ powders at the 10:5:1 molar ratio. Enhanced photoactivity could be related to the two-photon excitation in KTaO_3_-CdS-MoS_2_ composite under UV-Vis and/or to additional presence of CdMoO_4_ working as co-catalyst.

## 1. Introduction

Since the discovery of the photocatalytic splitting of water on TiO_2_ photochemical electrodes by Fujishima and Honda in 1972 [1], semiconductor-based photocatalysts have attracted increasing interest due to their potential applications in solar energy conversion, hydrogen evolution and photodegradation of organic pollutants [2,3,4,5,6,7,8,9,10,11]. Among different photocatalysts, titanium dioxide has become the most studied and widely used semiconductor material, however, it can only be excited by UV light, leading to low efficiency for utilizing solar energy [12]. Therefore, it is still of great importance to develop new types of photocatalysts which should be highly active, photostable and activated by low powered and low cost irradiation sources. Furthermore, one of the promising approaches is combining some semiconductors to form composites which can improve the efficiency of a photocatalytic system because of novel or enhanced properties that do not exist in individual components [13,14,15,16]. It was demonstrated that the Bi_2_WO_6_-based photocatalysts systems could be efficiently used in chemical synthesis and fuel production [7], whilst the graphene based composites seem to be a promising material for solar energy conversion and selective transformations of organic compounds [11]. Zhang *et al.* showed that the nanocomposites of TiO_2_-graphene exhibits much higher photocatalytic activity and stability than bare titanium dioxide toward the gas-phase degradation of benzene [17].

Among semiconductors, potassium tantalate with a wide band gap could be an interesting alternative to the well-known titanium dioxide. Tantalates possess conduction bands consisting of Ta5d orbitals located at a more negative position than titanates (Ti3d) or niobates (Nb4d). Therefore, the high potential of the conduction band of tantalates could lead to being more advantageous in the photocatalytic reaction [18]. It has been reported by Liu *et al.* that sodium and potassium tantalates showed higher activities for water splitting than those of niobates synthesized by hydrothermal route. Moreover, the highest photocatalytic activity was exhibited by NaTaO_3_ powder with cubic crystalline structure [19]. It was also observed that when NiO co-catalysts were loaded on the tantalate semiconductors, the photocatalytic activities were drastically increased [20]. Furthermore, there could be correlation between the photocatalytic activity of ABO_3_ tantalates and preparation method as well as their crystal structure. Lin *et al*. showed that sol gel sodium tantalate with monoclinic crystalline structures exhibited higher photoactivity for water splitting than that with orthorhombic structure and obtained by solid-state method [21]. In other study Torres-Martínez *et al.* prepared sodium tantalate samples doped with Sm and La using sol-gel or solid state reaction methods. The best half-life time (t_1/2_ = 65 min) of photocatalytic degradation of methylene blue under UV light was shown by NaTaO_3_ doped with Sm obtained by sol-gel technique and heat treated at 600 °C [22]. However, in the literature there is still not enough information about photocatalytic activity of tantalate for degradation of organic pollutants, especially potassium tantalate has received very little research attention in this area.

In contrast to potassium tantalate, cadmium sulfide is among the most exhaustively investigated semiconductors. It has a wide range of applications, including solar cells, optoelectronic devices, fluorescence probes, sensors, laser light-emitting diodes and photocatalysis [23,24,25]. Unfortunately, bare CdS photocatalyst has very low separation efficiency of photogenerated electron-hole pairs and undergoes photocorrosion which limit its practical application [26]. In order to improve photoactivity and to inhibit the photocorrosion, cadmium sulfide is usually coupled with other semiconductors, including CdS/TiO_2_ [27,28,29], CdS/ZnO [30,31], CdS/ZnS [32], CdS/WO_3_ [33] as well as CdS/MoS_2_. In particular, MoS_2_ semiconductor is an efficient co-catalyst. Moreover, the unique layered structure of MoS_2_ endows many important properties, such as anisotropy, chemical stability and anti-photo corrosion. [34]. There were few studies on the MoS_2_-CdS system obtained by impregnation [35], ball-milling combined calcination [36], electrodeposition and chemical bath deposition [37] orhydrothermal [38] methods for hydrogen production under visible light. Zong *et al.* showed that individual CdS and MoS_2_ particles were almost inactive in hydrogen evolution compared to the MoS_2_ particles deposited onto CdS. Moreover, researchers observed that the rate of hydrogen evolution on MoS_2_/CdS was higher than on CdS particles loaded with other catalysts such as Pt, Ru, Rh, Pd and Au. This result was explained by the better electron transfer between MoS_2_ and CdS [39].

Binary and ternary composites based on potassium tantalate, cadmium sulfide and molybdenum disulfide could be new examples of photoactive materials. In this study we obtained various combinations of KTaO_3_, CdS, MoS_2_ single semiconductors as well as their precursor solutions using calcination and hydro/solvothermal routes. The effect of the preparation method, type and amount of the composite components on the surface properties and photocatalytic activity in phenol degradation in the aqueous phase as well as activity and photostability in toluene degradation in the gas phase was investigated. Light emitting diodes (LEDs) were used in the gas phase measurements as a promising irradiation source, which allow to reduce power consumption and costs of photocatalytic processes.

## 2. Results and Discussion

### 2.1. BET Surface Area

The surface area and the pore volume of KTaO_3_, CdS, MoS_2_ semiconductors and their binary and ternary composites prepared by hydro/solvothermal as well as hydro/solvothermal mixed solutions and calcination methods are summarized in Table 1. The surface area of as-prepared samples fluctuated from less than 0.1 to 17.5 m^2^/g and was dependent on type and amounts of semiconductors as well as preparation methods. The pure CdS and MoS_2_ semiconductors had BET surface area about 1.2 and 1.8 m^2^/g, respectively. The CdS-MoS_2_ composites containing more or the same amounts of CdS as MoS_2_ caused an increase in surface area whereas in the presence of the excess MoS_2_ in the composite a drop in the surface area was observed as compared with pure semiconductors. The sample CdS-MoS_2_ 4-1 presented the highest surface area (about 11.1 m^2^/g) from among CdS-MoS_2_ composites. The KTaO_3_-based composites prepared by hydro/solvothermal methods had higher BET surface areas than composites which were calcined. Extremely small surface area and nearly zero pore volume were observed for the KTaO_3_-MoS_2_ 10-1_C and KTaO_3_-CdS-MoS_2_ 10-1-1_C samples. In the case of binary KTaO_3_-based composites it could be seen that potassium tantalate containing a small amount of MoS_2_ had lower surface area than potassium tantalate containing a small amount of CdS. Moreover, in the case of ternary composites it could be also observed that the high loading of CdS resulted in a significant increase in surface area. The pore volumes of obtained photocatalysts were very low and fluctuated from zero to 0.0088 cm^3^/g. It was observed that the pore volumes increased with increasing the surface area.

**Table 1:** Sample label, preparation method, BET surface area and pore volume of obtained single semiconductors and their composites.

Sample Label	KTaO_3_:CdS:MoS_2_/Molar Ratio	Preparation Method	BET Surface Area/[m^2^/g]	Pore Volume/[cm^3^/g]
KTaO_3_	1:0:0	hydrothermal	0.1	0.0001
CdS	0:1:0	solvothermal	1.2	0.0006
MoS_2_	0:0:1	hydrothermal	1.8	0.0009
CdS-MoS_2_ 5-1	0:5:1	solvothermal mixed solutions	5.0	0.0026
CdS-MoS_2_ 4-1	0:4:1	solvothermal mixed solutions	11.1	0.0056
CdS-MoS_2_ 1-1	0:1:1	solvothermal mixed solutions	10.4	0.0052
CdS-MoS_2_ 1-5	0:1:5	solvothermal mixed solutions	1.0	0.0006
KTaO_3_-CdS 10-1_MS	10:1:0	solvothermal mixed solutions	17.5	0.0088
KTaO_3_-CdS 10-1_C	10:1:0	hydro/solvothermal and calcination	0.4	0.0002
KTaO_3_-MoS_2_ 10-1_MS	10:0:1	hydrothermal mixed solutions	2.8	0.0014
KTaO_3_-MoS_2_ 10-1_C	10:0:1	hydrothermal and calcination	<0.1	~0
KTaO_3_-CdS-MoS_2_ 10-1-1_MS	10:1:1	solvothermal mixed solutions	4.0	0.0019
KTaO_3_-CdS-MoS_2_ 10-1-1_C	10:1:1	hydro/solvothermal and calcination	<0.1	~0
KTaO_3_-CdS-MoS_2_ 10-5-1_MS	10:5:1	solvothermal mixed solutions	10.3	0.0051
KTaO_3_-CdS-MoS_2_ 10-5-1_C	10:5:1	hydro/solvothermal and calcination	0.5	0.0003

### 2.2. XRD Analysis

**Figure 1:**
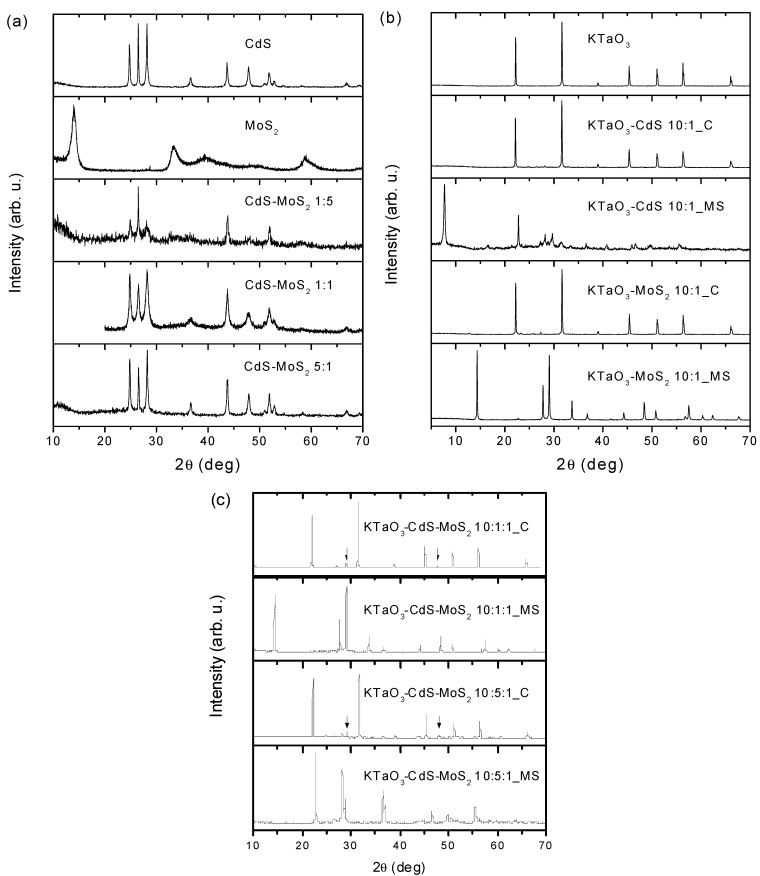
Powder XRD patterns for (**a**) single CdS, MoS_2_ and binary CdS-MoS_2_ nanocomposites, (**b**) single KTaO_3_ and binary KTaO_3_-based nanocomposites, (**c**) ternary KTaO_3_-based nanocomposites.

Figure 1a shows XRD patterns for pure CdS and MoS_2_ as well as CdS-MoS_2_ samples with different CdS:MoS_2_ molar ratio. Low observed intensity of the XRD peaks for MoS_2_ and for CdS-MoS_2_ with 1:5 ratio is likely caused by low crystallinity of the MoS_2_ compound. As can be seen, adding less than 20% of CdS to MoS_2_ causes a dramatic change in XRD pattern. CdS is a dominant phase and MoS_2_ is hardly present. No traces of MoS_2_ have been found for higher CdS concentration (CdS-MoS_2_ 1:1 and CdS-MoS_2_ 5:1 samples). Lattice parameters for CdS (*P*6_3_*mc*, s.g. #186) were refined by using the LeBail method.

For CdS we obtained a = 4.1341(8) Å and c = 6.710(1) Å in very good agreement with reported by Wiedemeire *et al.* [40]. Adding MoS_2_ does not change *a* lattice parameter, whereas *c* increases with increasing MoS_2_ and for the sample which contains the highest amount of MoS_2_ (sample b) the refined *c* is 6.733(3) Å. This clear change of the unit cell size and relative change of the intensity of three most dominant XRD peaks between 24 and 29 degrees, allow us to conclude that Mo atoms are incorporated into CdS hexagonal crystal structure. Figure 1b displays powder X-ray diffraction patterns for pure KTaO_3_ prepared by the hydrothermal method. Two different methods were used to obtain KTaO_3_-CdS and KTaO_3_-MoS_2_ composites. As can be seen from the XRD patterns for KTaO_3_-CdS 10:1_C and KTaO_3_-MoS_2_ 10:1_C samples, the calcination method does not produce new phases and KTaO_3_ together with CdS are observed. For the latest compound the most intense peaks are hardly visible between 25 and 30 deg. Contrary to the calcination method, the hydro/solvothermal mixed solutions route produces more complex compounds. For the system with CdS, the majority phase is Ta_2_O_5_ with small amount of TaS_2_ and K_2_Ta_15_O_32_. For the system with MoS_2_ almost pure pyrochlore-like K_2_Ta_2_O_6_ was found. This defect pyrochlore structure was described in details and also tested as a photocatalyst by Hu *et al.* [41].

Figure 1c shows XRD patterns for ternary KTaO_3_-CdS-MoS_2_ composites with two different molar ratios and two various preparation routes. Similarly to what was observed for binary composites produced by calcination method, the majority phase is KTaO_3_, however, a small amount of CdMoO_4_ emerges with the two most intense peaks marked by arrow. While the solvothermal mixed solutions method causes decomposition of KTaO_3_, the main products detected are K_2_Ta_2_O_6_ and Ta_2_O_5_ together with Mo-doped CdS for the sample 10:1:1 and 10:5:1, respectively.

### 2.3. Morphology

**Figure 2:**
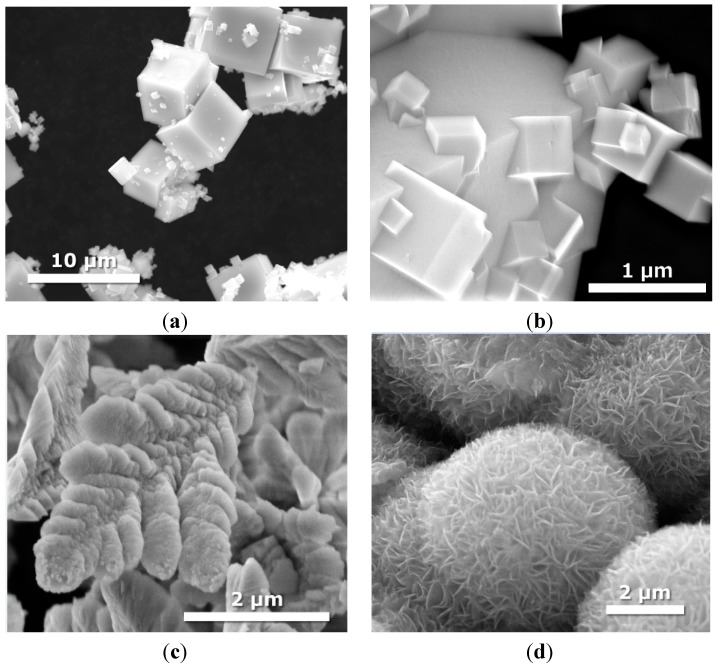
SEM images of single semiconductors obtained by hydrothermal method: (**a**,**b**) KTaO_3_; (**c**) CdS; (**d**) MoS_2_.

**Figure 3:**
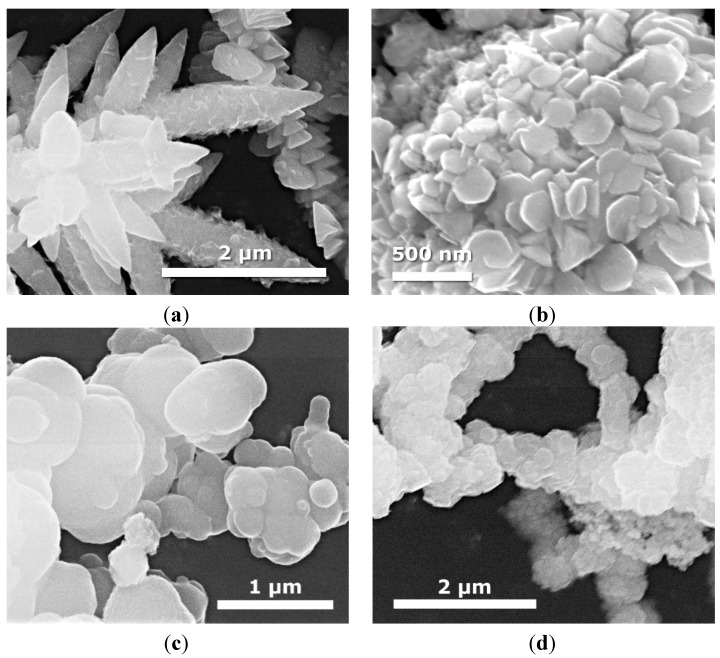
SEM images of binary CdS-MoS_2_ composites obtained by solvothermal mixed solution methods with different molar ratio of CdS: (**a**) CdS:MoS_2_ = 5:1; (sample CdS-MoS_2_ 5-1); (**b**) CdS:MoS_2_ = 4:1 (sample CdS-MoS_2_ 4-1); (**c**) CdS:MoS_2_ = 1:1 (sample CdS-MoS_2_ 1-1); and (**d**) CdS:MoS_2_ = 1:5 (sample CdS-MoS_2_ 1-5).

**Figure 4:**
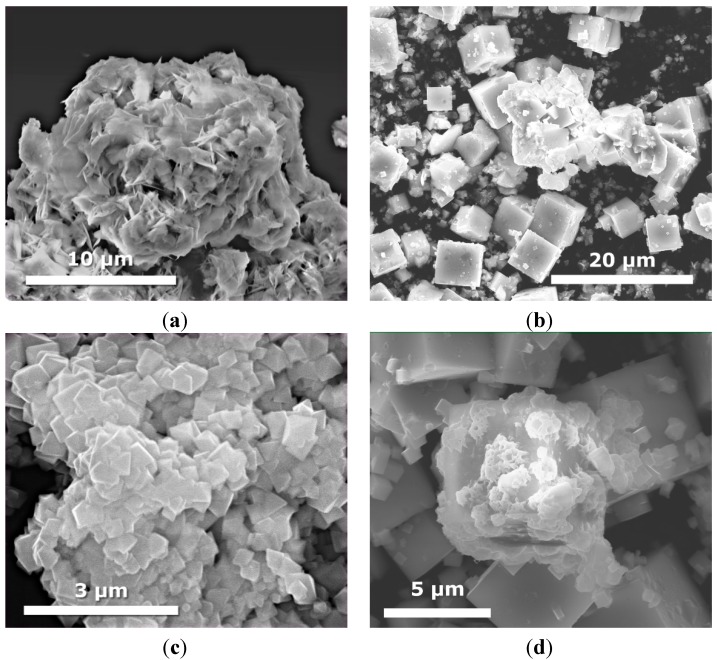
SEM images of binary KTaO_3_-CdS and KTaO_3_-MoS_2_ composites obtained with different molar ratio and using two preparation routes: (**a**) KTaO_3_-CdS (10:1) obtained by solvothermal mixed solutions (sample KTaO_3_-CdS 10-1_MS); (**b**) KTaO_3_-CdS (10:1) obtained by calcination of single previously synthesized semiconductors (sample KTaO_3_-CdS 10-1_C); (**c**) KTaO_3_-MoS_2_ (10:1) obtained by solvothermal mixed solutions (sample KTaO_3_-MoS_2_ 10-1_MS); and (**d**) KTaO_3_-MoS_2_ (10:1) obtained by calcination of single previously synthesized semiconductors (sample KTaO_3_-MoS_2_ 10-1_C).

**Figure 5:**
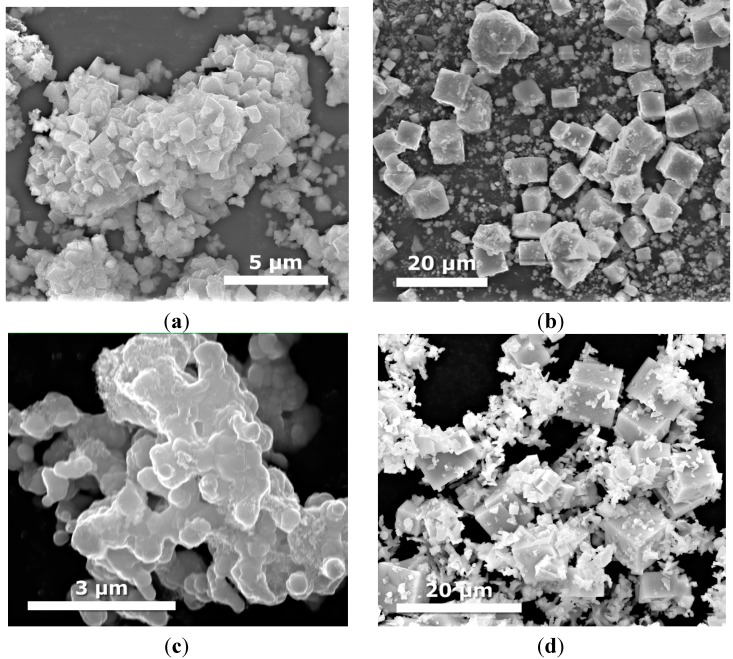
SEM images of ternary KTaO_3_-CdS-MoS_2_ composites obtained with different molar ratio and using two different preparation route: (**a**) KTaO_3_-CdS-MoS_2_ (10:1:1) obtained by solvothermal mixed solutions (sample KTaO_3_-CdS-MoS_2_ 10-1-1___MS); (**b**) KTaO_3_-CdS-MoS_2_ (10:1:1) obtained by calcination of single previously synthesized semiconductors (sample KTaO_3_-CdS-MoS_2_ 10-1-1___C); (**c**) KTaO_3_-CdS-MoS_2_ (10:5:1) obtained by solvothermal mixed solutions (sample KTaO_3_-CdS-MoS_2_ 10-5-1___MS); and (**d**) KTaO_3_-CdS-MoS_2_ (10:5:1) obtained by calcination of single previously synthesized semiconductors (sample KTaO_3_-CdS-MoS_2_ 10-5-1___C).

The morphology of the products was observed by SEM and is presented in the Figure 2, Figure 3, Figure 4 and Figure 5. Figure 2 shows an SEM image of single semiconductors prepared by hydro/solvothermal methods. As clearly shown, the KTaO_3_ have a cube-like shape with various sizes. Using higher magnification image of these particles, we notice that small KTaO_3_ nanocubes with an average size of about 0.2–1 µm grow at the surface of bigger cubes having widths of 5–8 µm. Pure CdS clearly indicates nanoleaf structures having the length of about 3 µm. In the case of MoS_2_ we observed that the product has the shape of flower-like microspheres with an average diameter of 5 µm.

Figure 3 shows SEM images of binary CdS-MoS_2_ composites with different CdS and MoS_2_ molar ratios. In the case of CdS-MoS_2_ 5-1 sample containing the highest molar ratio of CdS, we still observed nanoleaf structure indicating the presence of CdS. XRD analysis also showed that no traces of MoS_2_have been found for higher CdS concentration. The increase of MoS_2_ ratio resulted in the formation of hexagonal shaped nanostructures with average edge size of about 100–125 nm (Figure 3b).

As can be seen from Figure 3c,d, further increase in molar ratio of MoS_2_ to CdS causes a large change in microstructures. We can observe bonded structures of microspheres with diameters ranging from 0.08 to 1 µm.

Figure 4 displays SEM images of binary KTaO_3_-CdS and KTaO_3_-MoS_2_ composites prepared by two different routes. In the case of the KTaO_3_-CdS and KTaO_3_-MoS_2_samples, which were obtained by calcination, we can see mainly the structure of cubes, which indicates the presence of KTaO_3_. In contrast, the solvothermal mixed solutions route leads to the formation of other nanostructures, suggesting the presence of more complex compounds: K_2_Ta_15_O_32_, Ta_2_O_5_, TaS_2_ and K_2_Ta_2_O_6_ for the system with CdS and MoS_2_which were confirmed by XRD analysis. The transition of the KTaO_3_ structure from cubic to octahedral was observed for the samples KTaO_3_-MoS_2_ 10-1 and KTaO_3_-CdS-MoS_2_ 10-1-1 obtained by solvothermal mixed solutions. In the case of calcinated KTaO_3_-CdS-MoS_2_ 10-5-1 composite we can see nanoleaves of CdS deposited on the surface of large cubes of KTaO_3_ (Figure 5d). Contrary to calcination, solvothermal mixed solutions method causes dramatic changes in the structure—neither cubes nor nanoleaf are observed (Figure 5c).

### 2.4. UV-Vis Properties

**Figure 6:**
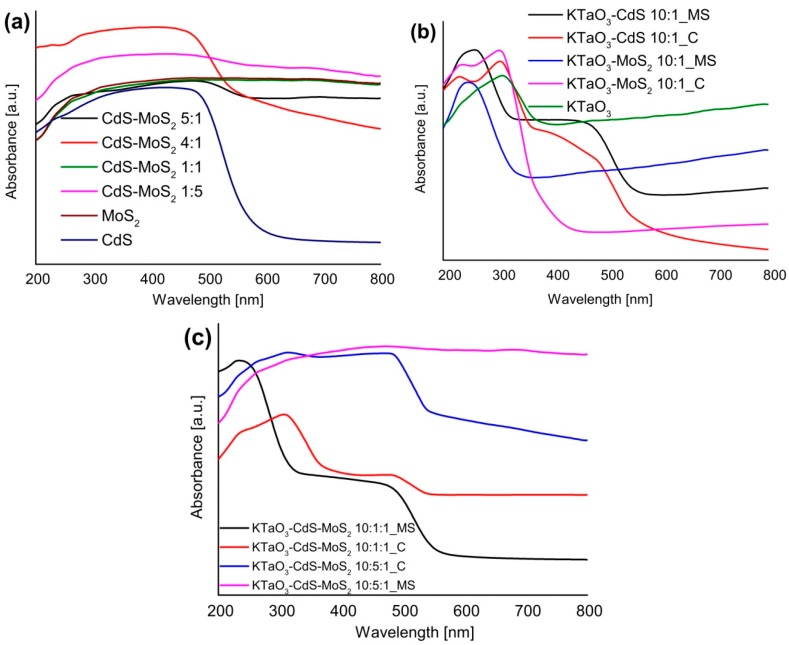
The UV-Vis diffuse reflectance spectra of (**a**) single CdS, MoS_2,_ and binary CdS-MoS_2_ nanocomposites, (**b**) single KTaO_3_ and binary KTaO_3_-based nanocomposites, (**c**) ternary KTaO_3_-based nanocomposites.

DRS UV-Vis absorption spectra in the wavelength range of 200–800 nm of as-prepared samples were investigated and the results are shown in Figure 6a–c. Figure 6a depicts the spectra for pure CdS and MoS_2_ semiconductors and their binary composites with varying molar ratio between CdS and MoS_2_. As it can be seen, all the samples could absorb both UV and visible light. The absorption edge of single CdS is at about 510 nm, which coincides with the literature. Moreover, it was previously reported that the absorption properties of CdS are strongly shape-dependent [42]. It could also be seen that the loading of MoS_2_ on CdS improved the light absorption as compared with pure CdS. Liu *et al.* also presented enhanced absorption properties for CdS-MoS_2_ composites to photocatalytic H_2_ production [38]. In comparison with single CdS, composites with excess of CdS exhibited a red-shift and a less steep absorption edge.

The absorbance was very similar for pure MoS_2_ and composite with 1:1 molar ratio between components. However, for composites containing excess MoS_2_, absorption was more intense. Figure 6b displays absorption spectra of single KTaO_3_ and binary KTaO_3_-based composites containing small amounts of CdS or MoS_2_ and prepared by two different procedures. In the case of single KTaO_3_, there is an obvious absorption band centered at about 310 nm and no absorption peak is detected above 310 nm. For octahedral KTaO_3_ nanocrystalline obtained by Zou *et al.* using hydrothermal method, the absorption peak was detected at 265 nm [43]. It was also observed that binary KTaO_3_-based composites loaded with small amount of CdS or MoS_2_ had steeper absorption edges and maximum absorption shifted to shorter wavelengths as compared with single potassium tantalate. Moreover, KTaO_3_-CdS composites showed second steep absorption edges in the visible light region. Figure 6c shows the spectra for ternary KTaO_3_-CdS-MoS_2_ composites containing different amount of CdS and prepared by various methods. Loading of larger amounts of CdS onto KTaO_3_-CdS-MoS_2_ composites significantly improved the spectral absorption. For ternary composites containing greater amount of cadmium sulfide and obtained by both methods, there were widest absorption ranges among all obtained samples but only for a calcined composite a steep absorption edge (at about 510 nm) was observed. In general, loading MoS_2_ onto CdS shifted absorbance peaks to longer wavelengths, just as loading CdS onto KTaO_3_ enhanced absorption features. It may be suggested that the best adsorption properties could probably be achieved for the ternary KTaO_3_-CdS-MoS_2_ composites containing appropriate molar ratio between semiconductors.

### 2.5. The Photocatalytic Degradation of Phenol in the Aqueous Phase

**Figure 7:**
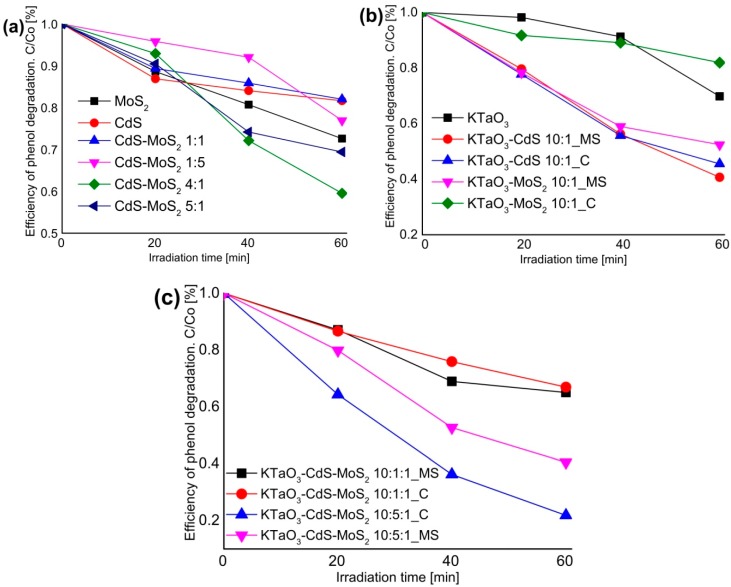
The percentage degradation of phenol at various time intervals under UV-Vis light in the presence: (**a**) single CdS, MoS_2_, and CdS-MoS_2_ nanocomposites; (**b**) single KTaO_3_ and binary KTaO_3_-based nanocomposites; (**c**) ternary KTaO_3_-based nanocomposites.

**Figure 8:**
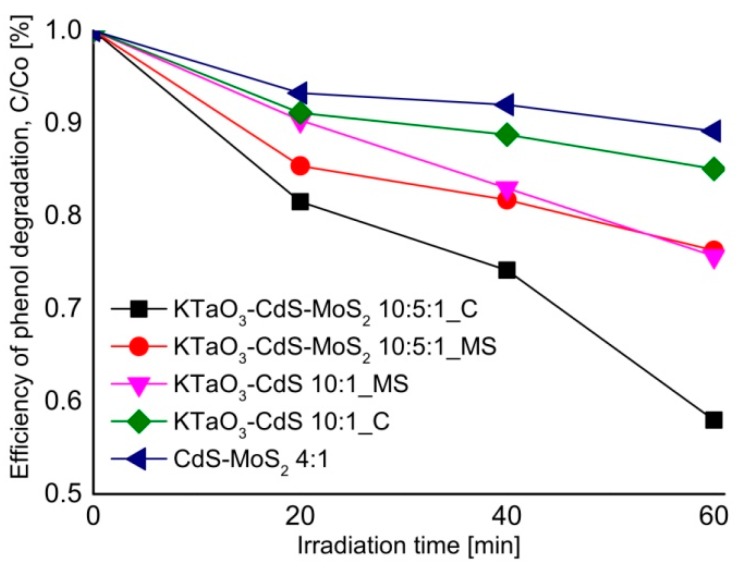
The percentage degradation of phenol at various time intervals under visible light in the presence of selected composites.

Photocatalytic activity of as-prepared semiconductors and their nanocomposites was estimated by examining the reaction of phenol degradation in the presence of UV-Vis light irradiation and for selected samples photoactivity under visible light was also analyzed. No degradation of phenol was observed in the absence of photocatalyst or illumination. Photocatalytic activity under UV-Vis and visible light is presented as phenol degradation rate (Table 2) and as efficiency of phenol removal after 60 min of irradiation (Figure 7 and Figure 8). Commercially available P25 TiO_2_ was used as a standard reference material in photocatalytic activity measurements.

Figure 7a shows the results of phenol degradation under UV-Vis light in the presence of pure CdS and MoS_2_, and their binary composites synthesized by hydro/solvothermal methods. In comparison with pure CdS and MoS_2_, photocatalytic activity for degradation of phenol increased in the presence of CdS-MoS_2_ nanocomposites containing excess of CdS. In the presence of CdS-MoS_2_ 4:1 and CdS-MoS_2_ 5:1, the percentage degradation of phenol after 60 min was about 41% and 31%, respectively, which indicates that with decreasing excess of CdS, the photocatalytic activity is relatively higher. For composites containing more or the same amount of MoS_2_ as CdS, photoactivity was comparable with pure semiconductors. The highest phenol degradation rate, about 1.41 µmol·dm^−3^·min^−1^, was observed in the case of the CdS-MoS_2_ 4:1 composite. For the other samples degradation rates were low and fluctuated from 0.62 to 0.90 µmol·dm^−3^·min^−1^. Zong and co-workers reported that the photocatalytic H_2_ production achieved a maximum when the loading amount of MoS_2_ on CdS was about 0.2 wt % [39]. Figure 7b displays the results of phenol degradation under UV-Vis light in the presence of single KTaO_3_ and binary potassium tantalate-based composites containing a small amount of CdS or MoS_2_ and prepared by different routes. The phenol degradation after 60 min of irradiation was only 18% in the case of the calcined KTaO_3_-MoS_2_ sample, which is lower than in the presence of single KTaO_3_ (about 30%). While for the KTaO_3_-MoS_2_ sample obtained by hydrothermal method phenol degradation was more efficient (about 48%). It could be also observed that photoactivities were similar and increased to 59% and 55% for KTaO_3_-CdS 10-1 composites prepared by hydrothermal and calcination method, respectively. It may be suggested that loading cadmium sulfide onto KTaO_3_ enhanced photoactivity, while the influence of introduction of molybdenum disulfide on the photoactivity was dependent on preparation technique. Figure 7c depicts the results of phenol degradation under UV-Vis light for ternary KTaO_3_-CdS-MoS_2_ composites containing various amounts of CdS as well as prepared by different methods. In general, more addition of CdS significantly enhanced the photocatalytic activity of ternary composites. In the presence of the KTaO_3_-CdS-MoS_2_ 10-1-1 sample the percentage degradation of phenol was low (about 35%) and quite comparable for both methods. In the case of the KTaO_3_-CdS-MoS_2_ 10-5-1 composites photoactivities were much more efficient. After 60 min of irradiation, for the sample prepared by hydro/solvothermal method phenol degradation reached 60%. At the same time in the presence of the KTaO_3_-CdS-MoS_2_ 10-5-1 sample prepared by the calcination method about 80% of phenol was degraded and it was the highest efficiency among all obtained samples. Therefore, appropriate ratio between KTaO_3_, CdS and MoS_2_ semiconductors in the composites is supposed to be responsible for the highest efficiency of phenol photodegradation. This deduction coincides with the UV-Vis properties of obtained samples described in Section 3.3.

Figure 8 shows the results of phenol degradation under visible light for selected samples which exhibited the highest efficiency of phenol degradation under UV-Vis light irradiation. Among these, the highest activity (about 42%) after 60 min of visible irradiation was also exhibited by calcined ternary KTaO_3_-CdS-MoS_2_ 10-5-1 composites. In the case of the binary calcined composite containing small amount of CdS, photoactivity reached about 15%. In the presence of samples obtained by the solvothermal mixed solutions route activity was more efficient (24%). The lowest activity (about 11%) was observed for the composite containing CdS and MoS_2_ at the 4:1 molar ratio.

### 2.6. The Photocatalytic Degradation of Toluene in the Gas Phase

The photocatalytic activity and stability of as-prepared photocatalysts were also evaluated by degradation of the toluene in the gas phase under UV light irradiation using low powered irradiation source, e.g., LEDs (λ_max_ = 375 nm) in four subsequent measurement cycles. The efficiency of toluene photodegradation after 60 min in the presence of as-prepared semiconductor samples is given in Table 2.

It could be seen that CdS, KTaO_3_ and MoS_2_ semiconductors and their composites were photoactive in toluene degradation. In the case of single semiconductors, removal efficiency in the presence of CdS reached about 57% after a 60-min process and activity was stable in four subsequent cycles of irradiation. While in the presence of KTaO_3_ and MoS_2_ photocatalytic activity decreased from 64% and 46% (1st cycle) to 37% and 22% (4th cycle), respectively. The highest activity and stability under UV light irradiation from all among CdS-MoS_2_ composites was exhibited by samples at the molar ratio 4:1 and 1:1.

**Table 2:** Photocatalytic activities of obtained samples for toluene photodegradation in four subsequent measurement cycles and for phenol degradation presented as phenol degradation rate.

Sample Label	Phenol Degradation Rate under UV-Vis/(µmol·dm^−^^3^·min^−^^1^)	Toluene Degradation after 1 h Irradiation (LEDs, λ_max_ = 375 nm) [%]			
		1st Cycle	2nd Cycle	3rd Cycle	4th Cycle
KTaO_3_	0.79	64	63	42	37
CdS	0.61	57	57	57	52
MoS_2_	0.90	46	23	22	22
CdS-MoS_2_ 1-5	0.77	57	53	44	27
CdS-MoS_2_ 1-1	0.62	61	53	62	52
CdS-MoS_2_ 5-1	0.81	70	60	49	48
CdS-MoS_2_ 4-1	1.41	53	56	60	62
KTaO_3_-CdS 10-1_MS	2.08	47	45	41	40
KTaO_3_-CdS 10-1_C	1.75	53	48	52	50
KTaO_3_-MoS_2_ 10-1_MS	1.69	55	51	49	51
KTaO_3_-MoS_2_ 10-1_C	0.55	46	34	37	35
KTaO_3_-CdS-MoS_2_ 10-1-1_MS	1.15	50	52	48	39
KTaO_3_-CdS-MoS_2_ 10-1-1_C	1.11	53	54	49	40
KTaO_3_-CdS-MoS_2_ 10-5-1_MS	1.99	50	41	43	41
KTaO_3_-CdS-MoS_2_ 10-5-1_C	2.81	48	48	50	46
TiO_2_ (P25)	2.87	98	96	95	95

The lowest photostability presented the CdS-MoS_2_ sample when the molar ratio was 1:5. It suggests that an excess of MoS_2_ caused the decrease in photostability. The CdS-MoS_2_ composite at the molar ratio 5:1 exhibited the highest activity in the 1st cycle (about 70%) but this activity decreased to 48% in the 4th cycle of photoirradiation. In the case of binary KTaO_3_-based composites it was observed that the photoactivity about 50% and the best stability during four cycles of irradiation was exhibited by potassium tantalate with a small amount of cadmium sulfide (KTaO_3_-CdS 10-1 sample) prepared by the calcination method and potassium tantalate with molybdenum disulfide (KTaO_3_-MoS_2_ 10-1 sample) obtained by the hydrothermal mixed solutions route. While the photocatalytic activity of the KTaO_3_-MoS_2_ 10-1 sample prepared by calcination method after four cycles of irradiation was less by 11% as compared with the first cycle. It indicates that the activity is dependent not only on the type and content of the composite components but also on the preparation method. All of prepared ternary KTaO_3_-CdS-MoS_2_ composites exhibited about 50% efficiency degradation of toluene after the 1st cycle of irradiation, but activity didn't change after four cycles only in the case of the sample KTaO_3_-CdS-MoS_2_ 10-5-1 obtained by the calcination method.

### 2.7. The Origin of Photocatalytic Activity

**Figure 9:**
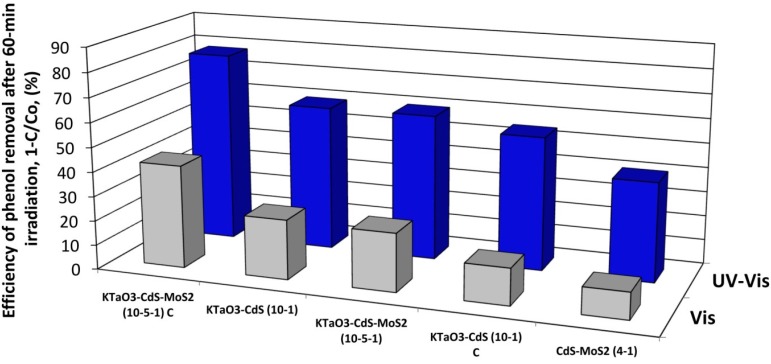
Efficiency of phenol removal under UV-Vis and visible light (λ > 420 nm) for selected binary and ternary composites.

**Figure 10:**
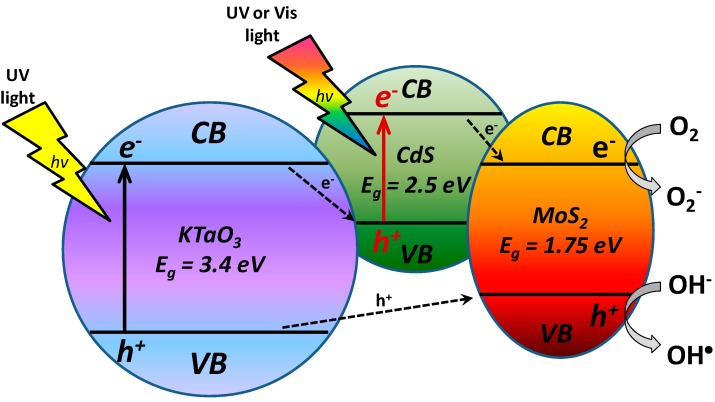
Probable UV-Vis two-photon excitation cycle of KTaO_3_-CdS-MoS_2_ composite: (1) UV induced excitation in solid KTaO_3_ (*E_g_* = 3.4 eV) to generate free electrons (*e^−^*) and holes (*h^+^*), and since the VB position of KTaO_3_ is lower than the VB band of MoS_2_, *h^+^* transfer would then occur from KTaO_3_ to MoS_2_; (2) UV or visible light excitation of solid CdS (*E_g_* = 2.5 eV) also generates *e^−^* and *h^+^* in CdS, and as the CB position of CdS is higher in energy than the CB of solid MoS_2_, electron transfer could occur from CdS to MoS_2_, thereby causing a charge separation at the CdS-MoS_2_ junction; (3) positioning the CB of KTaO_3_ higher in energy than the VB of CdS causes *e^−^* transfer from KTaO_3_ to CdS and recombination of *e^−^* in KTaO_3_ with h+ generated in CdS, thus completing the photoexcitation cycle in which the excited state of MoS_2_ has been achieved via a two-photon process.

UV and visible light induced photoactivity presented as efficiency of phenol removal after 60-min irradiation for the most active binary and ternary composites is shown in Figure 9. Highest activity under UV-Vis light of KTaO_3_-CdS-MoS_2_ (10:5:1) composite, obtained by solvothermal preparation of single semiconductors followed by calcination, could be related to the presence of heterojunctions between semiconductors. One pot reaction used for preparation of the KTaO_3_-CdS-MoS_2_ composite did not allow the formation of well-developed crystal structure of all semiconductors, as was shown in XRD analysis. However, the structure obtained by the sintering of three components could favors photogenerated charge carriers transfer between phase boundary of semiconductors resulted in higher photoactivity. The KTaO_3_-CdS-MoS_2_ nanocomposite could be excited by two photons due to utilization of a heterojunction between KTaO_3_ with two low band gap semiconductors (*i.e.*, CdS and MoS_2_). As shown in Figure 10, UV light could induce excitation in solid KTaO_3_ (*E_g_* = 3.4 eV) and generate free electrons (*e^−^*) and holes (*h^+^*). Since the VB position of KTaO_3_ (+3.1 V) is lower than the VB band of MoS_2_ (+1.8 V), *h^+^* transfer would then occur from KTaO_3_ to MoS_2_. At the same time UV or visible light could be absorbed by CdS (*E_g_* = 2.5 eV) and generate *e^−^* and *h^+^* in CdS. As the CB position of CdS (−0.6 V) is higher in energy than the CB of solid MoS_2_ (+0.05 V), electron transfer could occur from CdS to MoS_2_, thereby causing charge separation at the CdS-MoS_2_ junction. Finally positioning the CB of KTaO_3_ (−0.3 V) higher in energy than the VB of CdS (+1.8 V) causes *e^−^* transfer from KTaO_3_ to CdS and recombination of *e^−^* in KTaO_3_ with *h^+^* generated in CdS, thus completing the photoexcitation cycle in which the excited state of MoS_2_ has been achieved via a two-photon process. Thus, organic compounds (such as phenol or toluene) could be degraded via active oxygen species generated at the surface of excited MoS_2_ in two photons process.

Additionally, higher activity under UV-Vis light of the KTaO_3_-CdS-MoS_2_ 10-5-1_C sample could be also related to the presence of CdMoO_4_ detected by XRD analysis (see Figure 1c). Photocatalytic activity of pure and *Ag*-modified CdMoO_4_ under simulate solar irradiation was reported by others [44,45]. The valence band of CdMoO_4_ consists of the hybrid orbitals of O 2p as well as Cd 6s and the conduction band consists of Mo 4d orbital and the band gap energies between them is about 3.4 eV. Thus, CdMoO_4_ presented at the surface of composite could also adsorb UV light and enhance activity in observed photodegradation reaction. Lower UV and Vis-induced photoactivity of KTaO_3_-CdS-MoS_2_ (10:5:1) composite obtained by mixed solvothermal reaction, comparing to that one prepared by calcination step, could be related the decomposition of KTaO_3_ and formation of K_2_Ta_2_O_6_ and Ta_2_O_5_ as main component of as-prepared samples.

## 3. Experimental

### 3.1. Materials and Instruments

Tantalum (V) oxide (>99% Aldrich, Poznan, Poland) and potassium hydroxide (Chempur, pure p.a.) were used as precursors for the preparation of KTaO_3_. Thiourea (Aldrich, 99%) has been chosen as a sulfur source to synthesize sulfides. CdCl_2_·2H_2_O (Sigma-Aldrich, ≥99%) and Na_2_MoO_4_·2H_2_O (Sigma-Aldrich, ≥99.5%) were used as precursors of Cd and Mo, respectively. Ethanol, polyethylene glycol 400 (PEG-400), ethylene glycol (EG*)* were purchased from POCH S.A. (Gliwice, Poland) and used without further purification. Deionized water was used for all reactions and treatment processes. A commercial form of TiO_2_ (P25, crystalline composition: 80% anatase, 20% rutile, surface area 50 g/m^2^) from Evonik (Essen, Germany) was used for the comparison of the photocatalytic activity.

Nitrogen adsorption–desorption isotherms at 77 K were measured using a Micromeritics Gemini V (model 2365) physisorption analyzer (Micromeritics Instrument, Norcross, GA, USA). Specific surface areas were calculated following typical Brunauer–Emmett–Teller (BET) method using the adsorption data in the relative pressure (p/p_0_) range from 0.05 to 0.3. Prior to adsorption measurements the samples were degassed under vacuum at 200 *°*C for 2 h.

Diffuse reflectance spectra (DRS) of the synthesized materials were characterized using the Thermo Scientific Evolution 220 UV-Visible spectrophotometer (Thermo Scientific, Waltham, MA, USA) equipped with ISA-220 integrating sphere accessory. The UV-Vis DRS spectra were recorded in the range of 200–800 nm using a barium sulfate reference.

Powder X-ray diffraction (PXRD, Philips/PANalytical X'Pert Pro MPD diffractometer, (PANalytical, Almelo, The Netherlands) with Cu K_α_ radiation λ = 1.5418 Å) was used to determine the phase composition and calculate lattice parameters of polycrystalline samples.

The morphology of the semiconductor composites was investigated with FEI Quanta 250 FEG scanning electron microscope (SEM; FEI, Hillsboro, OR, USA) working in high vacuum mode. Energy-dispersive X-ray spectroscopy (EDS) measurements were carried out using SEM-integrated EDAX Apollo-SDD detector (EDAX Inc., Mahwah, NJ, USA). Accelerating voltage was set to 30 kV. Standardless analysis was conducted with the EDAX TEAM software with *eZAF* quantization method.

### 3.2. Synthesis of Single KTaO_3_, CdS and MoS_2_ Semiconductors

KTaO_3_, CdS and MoS_2_ semiconductors were prepared by hydro/solvothermal methods using an autoclave with a capacity of 500 mL as the reactor. In a typical procedure for the preparation of potassium tantalate, KOH (30 g) was dissolved in deionized water (60 mL), then Ta_2_O_5_ (11 g) and PEG-400 (3 mL) were added. This mixture (marked as *solution A*) was stirred for 1 h before it was transferred into a Teflon-lined stainless steel autoclave. The autoclave was sealed and got heated at 200 *°*C for 24 h. After cooling naturally to room temperature, the resulting powder was washed several times by centrifugation with distilled water and ethanol respectively and dried in an oven at 70 *°*C for 8 h. Finally, some white powder was obtained.

Cadmium sulfide was prepared based on the methodology presented by Zhong *et al.* [42]. In a typical procedure, CdCl_2_ (18.3 g) was added into deionized water (160 mL) and EG (160 mL) under continuous stirring for 15 min. After that, thiourea (15.2 g) was added to the solution and stirring was continued for an additional 20 min*.* This mixture (marked as *solution B*) was transferred into the autoclave which was sealed and maintained at 190 *°*C for 24 h and allowed to cool down to room temperature naturally. The crystalline powder product was washed several times by centrifugation with distilled water and ethanol, respectively and dried in the oven at 70 *°*C for 8 h. Finally, some yellow powder was obtained.

Molybdenum disulfide was synthesized using a method similar to reported by Wang *et al.* [46]. To prepare MoS_2_, Na_2_MoO_4_·2H_2_O (12.1 g), thiourea (15.6 g) and PEG-400 (3.5 g) were dissolved in deionized water (300 mL). The solution was stirred continuously for 15 min. The resulting mixture (marked as *solution C*) was transferred into the teflon-lined stainless autoclave which was maintained at 200 *°*C for 24 h, then cooled to room temperature. The precipitate was washed with deionized water and dried in the oven at 80 *°*C for 6 h. Finally, some black powder was obtained.

### 3.3. Synthesis of Binary and Ternary Semiconductor Composites

The KTaO_3_, CdS and MoS_2_ powders or solutions A, B, C prepared according to the methods described in Section 2.2 were combined to form semiconductor nanocomposites by the calcination or by the hydro/solvothermal mixed solutions methods, respectively. All prepared samples with different molar ratios between semiconductors are presented in Table 1.

To prepare KTaO_3_-CdS, KTaO_3_-MoS_2_ and KTaO_3_-CdS-MoS_2_ composites by the calcination method, KTaO_3_ and CdS or/and MoS_2_ powders, respectively were mixed together sufficiently with various molar ratios. The powder mixture was calcined in the oven at 500 °C for 3 h with a heating rate of 3 °C/min. Then the heated mixture was removed from the oven and allowed to cool to room temperature naturally.

A typical hydro/solvothermal mixed solutions process for the preparation of CdS-MoS_2_; KTaO_3_-CdS; KTaO_3_-MoS_2_; KTaO_3_-CdS-MoS_2_ composites with various molar ratios between semiconductors was as follows: the required amounts of solutions B and C; A and B; A and C; A and B and C, respectively were mixed together and stirred continuously for 45 min using a magnetic stirrer. Then the as-prepared mixture was placed in the autoclave and heated at 200 *°*C for 24 h. The resulting precipitate was washed with distilled water and ethanol, respectively and dried in the oven at 70 *°*C for 8 h.

### 3.4. Measurement of Photocatalytic Activity in the Aqueous Phase

The photocatalytic activity of KTaO_3_, CdS and MoS_2_ single semiconductors and their nanocomposite powder in ultraviolet (UV) and visible light (Vis) was estimated by monitoring the decomposition rate of 0.21 mM phenol in the aqueous solution. Phenol was selected as a model contaminant because it is a non-volatile and common organic pollutant found in various types of industrial wastewater. Photocatalytic degradation runs were preceded by blind tests in the absence of a photocatalyst or illumination. The aqueous phase containing the photocatalyst (125 mg), deionized water (24 mL) and phenol (1 mL, C = 500 mg/dm^3^) was placed in a photocatalytic reactor (V = 25 cm^3^) equipped with a 30 mm-thick quartz window. The temperature of the aqueous phase during the experiments was maintained at 10 °C by an external circulating water bath. The prepared suspension was stirred using magnetic stirrer and aerated (V = 5 dm^3^/h) for 30 min in the dark to reach the adsorption equilibrium and then the content of the reactor was photoirradiated with a 1000 W Xenon lamp (Oriel Instruments, Stratford, CT, USA) which emitted both UV and Vis irradiation. The optical path included a water filter and glass filters (GG420, Optel, Opole, Poland) to cut off IR and/or UV, respectively. GG glass filter transmitted light of wavelength greater than 420 nm. During the irradiation, 1 cm^3^ of suspension sample was collected at regular time periods and filtered through syringe filters (Ø = 0.2 µm) to remove the photocatalyst particles. Phenol concentration was estimated by colorimetric method after derivatization with diazo-*p*-nitroaniline using UV-Vis spectrophotometer (DU-7, Beckman, Warsaw, Poland).

### 3.5. Measurement of Photocatalytic Activity in the Gas Phase

The photocatalytic activity of the prepared semiconductors and their nanocomposite powders was also determined in the toluene degradation process. Toluene, an important volatile organic compound (VOC), was used as a model air contaminant. The photocatalysts activity tests were carried out in the flat stainless steel reactor (V = 30 cm^3^) equipped with a quartz window, two valves and a septa. As an irradiation source there was used an array of 25 LEDs (λ_max_ = 375 nm, 63 mW per diode) which was described in our previous study [47]. In a typical measurement the semiconductor powder (about 0.1 g) was suspended in a small amount of water and loaded as a thick film on a glass plate (3 cm × 3 cm) using painting technique. The obtained semiconductors coated support was dried and then placed at the bottom side of the photoreactor followed by closing the reactor with a quartz window. The gaseous mixture from a cylinder was passed through the reactor space for 1 min. The concentration of toluene in a gas mixture was about 150 ppm. After closing the valves, the reactor was kept in the dark for 15 min to reach adsorption equilibrium. A reference sample was taken just before starting irradiation. To estimate toluene concentration the samples were taken every 10 min during 60 min of irradiation. The photocatalytic stability was estimated in four subsequent cycles of toluene degradation. The analysis of toluene concentration in the gas phase was carried out using a Perkin Elmer Clarus 500 GC (Perkin Elmer, Waltham, MA, USA) equipped with a 30 m × 0.25 mm Elite-5 MS capillary column (0.25 μm film thickness) and a flame ionization detector (FID). The samples (200 µL) were injected by using a gas-tight syringe. Helium was used as a carrier gas at a flow rate of 1 mL*/*min.

## 4. Conclusions

A series of novel binary and ternary composite photocatalysts was obtained based on the combination of potassium tantalate, cadmium sulfide and molybdenum disulfide powders as well as their hydro/solvothermal precursor solutions. It was observed that calcination is an effective method to prepare various KTaO_3_, CdS and MoS_2_ semiconductor combinations, the while solvothermal mixed precursor solutions route led to obtaining other composite components. The UV-Vis DRS spectra showed that the loading MoS_2_ onto CdS shifted absorbance peaks to longer wavelengths as well as loading CdS onto KTaO_3_ enhanced absorption properties. On the other hand, an excess of MoS_2_ in composite caused a decrease in activity both for phenol and toluene degradation. It may be suggested that the best absorption properties and the highest activity could probably be achieved for the ternary composites containing appropriate molar ratio between above semiconductors but it needs further investigation. We found that the ternary semiconductor hybrid prepared by calcination of KTaO_3_, CdS and MoS_2_ powders at the 10:5:1 molar ratio exhibited very good stability during four measurement cycles in toluene degradation and excellent photocatalytic performance in phenol degradation among all obtained photocatalysts–the activity reached 80% under UV-Vis and 42% under Vis light 60-min irradiation. This relatively high photoactivity under visible light could be related to the presence of heterojunction between semiconductors. Due to the band structure of component semiconductors, two-photon excitation together with charge carrier transfer between KTaO_3_, CdS and MoS_2_ could be expected. However, ternary composite with 10:1:1 molar ratio between semiconductors exhibited relatively low activity which proved that the photocatalytic efficiency in aqueous phase reaction strictly depends on amount of composite components and need further investigation. In general, this work demonstrates that novel composites based on KTaO_3_, CdS and MoS_2_ are promising as photocatalysts for the degradation of organic pollutants in both the gas and aqueous phases*.*
